# Post-retrieval Extinction Prevents Reconsolidation of Methamphetamine Memory Traces and Subsequent Reinstatement of Methamphetamine Seeking

**DOI:** 10.3389/fnmol.2019.00157

**Published:** 2019-07-02

**Authors:** Ya-Yun Chen, Li-Bo Zhang, Yue Li, Shi-Qiu Meng, Yi-Miao Gong, Lin Lu, Yan-Xue Xue, Jie Shi

**Affiliations:** ^1^Department of Pharmacology, School of Basic Medical Sciences, Peking University Health Science Center, Beijing, China; ^2^Beijing Key Laboratory of Drug Dependence, National Institute on Drug Dependence, Peking University, Beijing, China; ^3^Peking-Tsinghua Center for Life Sciences and PKU-IDG/McGovern Institute for Brain Research, Peking University, Beijing, China; ^4^Peking University Sixth Hospital/Peking University Institute of Mental Health, Peking University, Beijing, China; ^5^National Clinical Research Center for Mental Disorders (Peking University Sixth Hospital), Beijing, China

**Keywords:** methamphetamine, retrieval, extinction, relapse, amygdala

## Abstract

Methamphetamine abuse has become a serious public health problem. However, effective treatment for methamphetamine addiction remains elusive, especially considering its high rate of relapse after treatment. A conditioned stimulus (CS) memory retrieval–extinction procedure has been demonstrated to decrease reinstatement of cocaine, heroin, and alcohol seeking in rats, and to reduce cue-induced cravings in heroin and nicotine addicts. The goal of the present study is to explore the effect of the CS memory retrieval–extinction procedure on methamphetamine seeking in rats and the underlying mechanisms. We found that daily retrieval of methamphetamine-associated memories 1 h before extinction sessions decreased subsequent drug priming-induced reinstatement, spontaneous recovery, and renewal of methamphetamine seeking. We also found that retrieval of methamphetamine-associated memories induced neuronal activation in the basolateral amygdala (BLA), while presenting extinction within the time window of reconsolidation abolished the neuronal activation in BLA. These results indicate that the CS memory retrieval–extinction procedure could prevent reconsolidation of methamphetamine memory traces in BLA and subsequent methamphetamine craving and relapse.

## Introduction

Methamphetamine addiction remains a significant public health concern worldwide, leading to devastating personal and social consequences. Frequent use of methamphetamine has been associated with severe neurotoxic effects and neurocognitive impairment (Ernst et al., 2000; Berman et al., 2008; Hart et al., 2012; Dean et al., 2013). Nevertheless, there has been no medication approved by the FDA for the treatment of methamphetamine addiction so far. Furthermore, psychosocial interventions, such as cognitive behavioral therapy, are considered to be cost- and time-intensive and with relatively poor outcomes given the high rates of relapse among methamphetamine addicts (Rawson et al., 2004; Shearer, 2007). Therefore, it is of great importance to develop more effective interventions to treat methamphetamine addiction and to prevent relapse.

Drug addiction has been considered to be a kind of aberrant reward memory (Kauer and Malenka, 2007). Associations between drug (unconditioned stimulus, US) and drug-related cues (conditioned stimulus, CS) play an important role in drug addiction and relapse (Stewart et al., 1984). In both animal and human studies, continuous exposure to drug-associated cues has been suggested to induce extinction and thus suppress drug craving and relapse. This effect has laid the theoretical foundations for exposure therapy to treat drug addiction in the clinic (Marlatt, 1990). However, the efficacy of exposure therapy is not persistent. Relapse can be induced by exposure to the drugs of abuse (reinstatement), drug-associated contexts (renewal), or the extension of withdrawal time after extinction (spontaneous recovery) (Bouton, 2002; Conklin and Tiffany, 2002).

After acquisition and stabilization, a consolidated memory can enter a labile state within a specific time window after reactivation and thus susceptible to enhancement or impairment, a process defined as reconsolidation (Nader et al., 2000; Alberini, 2005; Sorg, 2012; Reichelt and Lee, 2013). A number of studies have shown that drug-paired CS retrieval followed by pharmacological intervention could disrupt the reconsolidation of drug memories and inhibit drug conditioned place preference (CPP) and operant drug seeking and relapse (Li et al., 2010; Sanchez et al., 2010). However, most compounds used in these studies are not approved for human use except for the beta-adrenoceptor antagonist propranolol (Fricks-Gleason and Marshall, 2008; Wouda et al., 2010), and this may be an obstacle to successful translation to clinical applications.

In recent years, a CS memory retrieval–extinction procedure has been proposed as a nonpharmacological alternative to prevent reemergence of fear in rats and humans (Monfils et al., 2009; Schiller et al., 2010). When applying this CS memory retrieval–extinction procedure to drug addiction, we found that it can decrease drug-priming-induced reinstatement, renewal, and spontaneous recovery of drug (morphine, heroin, and cocaine) seeking in rats as well as cue-induced craving in heroin addicts (Xue et al., 2012). Consistent with our results, the CS memory retrieval–extinction procedure was also effective for reinstatement of morphine CPP and alcohol seeking in rats and cue-induced craving in smokers (Ma et al., 2012; Millan et al., 2013; Germeroth et al., 2017). However, the mechanisms underlying the inhibitory effect of the procedure on drug seeking have not yet been fully understood.

A large body of evidence indicates that the amygdala plays a pivotal role in retrieval, extinction, and reconsolidation of fear and drug memory (Nader et al., 2000; Lee et al., 2006; Milton et al., 2008a; Li et al., 2010; Luo et al., 2013; Xue et al., 2014), which suggested that amygdala may be implicated in the effects of CS memory retrieval–extinction. In addition, some studies have suggested that dephosphorylation of glutamate receptor 1 (GluA1), trafficking of calcium-permeable α-amino-3-hydroxyl-5-methyl-4 isoxazole-propionate receptors (CP-AMPARs), activation of L-type voltage-gated calcium channels, and the expression of immediate early gene, zinc-finger 268 protein (Zif268), and Arc may underlie the effects of the retrieval–extinction procedure (Monfils et al., 2009; Clem and Huganir, 2010; Flavell et al., 2011; Tedesco et al., 2014; Lee et al., 2016). However, few studies showed the neural substrate for the inhibitory effect of CS memory retrieval–extinction on drug seeking. Our previous study revealed that the CS memory retrieval–extinction procedure with a 10-min but not 6-h interval amplified the decrease in PKMζ expression in the basolateral amygdala (BLA) induced by extinction training (Xue et al., 2012). In the present study, we sought to identify whether the effects of the CS memory retrieval–extinction procedure can be extended to methamphetamine seeking and relapse, and we also explored the distinct neural activation patterns in the amygdala during different retrieval–extinction manipulations.

## Materials and Methods

### Subjects

Male Sprague–Dawley rats, weighing 260–280 g, were purchased from the Vital River Company. The rats were housed five per cage before the experiments and were individually housed after the surgery. The rats were maintained under controlled temperature (23 ± 2°C) and humidity (50 ± 5%) with free access to chow and water and were kept on a reverse 12-h light/dark cycle. The behavioral experiments were conducted during the dark phase of the cycle. The experimental procedures were performed in accordance with the National Institutes of Health Guide for the Care and Use of Laboratory Animals and were approved by the Biomedical Ethics Committee of animal use and protection of Peking University.

### Surgery

Rats were anesthetized with sodium pentobarbital (60 mg/kg, i.p.). Silastic catheters were inserted into the right jugular vein with the tip terminating at the opening of the right atrium based on our previous studies (Xue et al., 2012; Luo et al., 2015). All rats were allowed to recover from the surgery for 5–7 days.

### Intravenous Methamphetamine Self-administration Training

The procedure for methamphetamine self-administration was based on previous studies (Caprioli et al., 2015; Venniro et al., 2017). The operant chambers (AniLab Software & Instruments, Ningbo, China) had two nosepoke operandi located 5 cm above the bottom of the chambers. Nosepokes in the active operandum resulted in methamphetamine infusions and a 5-s tone-light cue. Nosepokes in the inactive operandum cannot lead to methamphetamine infusions or tone-light cues but were also recorded. The rats were trained to self-administer methamphetamine (0.10 mg/kg/infusion) during three 1-h sessions (separated by 5-min off periods) over 14 days. The self-administration training started at the beginning of the dark cycle and was performed under a fixed-ratio one (FR1) 40-s timeout-reinforcement schedule. Sessions began with the presentation of a houselight that remained on for the duration of the session. To prevent overdose, the number of methamphetamine infusions was limited to 15 per hour. After the self-administration training, rats were divided into different groups with matched methamphetamine intake during training phase.

### CS Memory Retrieval

The CS memory retrieval manipulation was based on our previous studies (Xue et al., 2012; Luo et al., 2015). The rats were given 15-min daily sessions during which nosepoke responses led to the 5-s tone-light cue but not methamphetamine infusions. The 180-min daily extinction sessions began 1 h or 6 h after the CS retrieval manipulation.

### Extinction

During the extinction sessions (195 min for the “no retrieval + extinction” group and 180 min for the “CS retrieval + extinction” group), the conditions were identical to that during training, with the exception that nosepoke responses led to the 5-s tone-light cue but not methamphetamine infusions. The rats underwent extinction training until their nosepokes on the active operandum were less than 20% of the mean nosepokes during the last 3 days of methamphetamine self-administration training for at least two consecutive days.

### Test for Drug Seeking

Once nosepokes on the active operandum was successfully extinguished according to the criteria described above, test for methamphetamine seeking began. The testing conditions were identical to that of training phase except for the fact that active nosepokes did not lead to methamphetamine infusions. The test session started with the presentation of the houselight that remained on throughout the test. Nosepoke responding during the test led to contingent presentations of the 5-s tone-light cue that had previously been paired with methamphetamine infusions. During the drug-priming-induced reinstatement tests, the rats were given an intraperitoneal injection of methamphetamine (1 mg/kg) immediately before the sessions began. The dose of priming was based on previous studies (Cox et al., 2013; Jing et al., 2014; Baracz et al., 2016).

### Immunofluorescence Staining and Imaging Analysis

Immunofluorescence assays were performed to examine Fos expressions in brain slices based on our previous studies (Xue et al., 2017; Fang et al., 2018). Rats were anesthetized with 10% chloral hydrate and perfused transcardially with 0.01 M phosphate buffered saline (PBS) followed by 4% paraformaldehyde (PFA). Brains were dissected and post-fixed in 4% PFA before being transferred to 30% sucrose in PB at 4°C. Then, the brains were frozen in dry ice and stored at −80°C until sectioning. Coronal sections of the amygdala were cut with a Leica cryostat at 20 μm thickness and washed with PBS three times for 5 min each before being incubated in PBS containing 0.3% Triton X-100 and 2% bovine serum albumin (BSA) for 1 h at 37°C. The sections were then incubated with rabbit antibody to Fos (1:500, #2250s, Cell Signaling Technology, Danvers, MA, USA) and mouse antibody to NeuN (1:500, #MAB377, Millipore, Burlington, MA, USA) for 24 h at 4°C. After incubation with the primary antibodies, sections were rinsed with PBS four times for 5 min each and then incubated with secondary antibodies (Alexa Fluor 488-conjugated goat anti-rabbit, 1:500, #A11034, Invitrogen; Alexa Fluor 594-conjugated donkey anti-mouse, 1:500, #A21203, Invitrogen) for 3 h at room temperature. After incubation with the secondary antibodies, the sections were rinsed four times for 5 min each, mounted, and coverslipped. Images were acquired by a fluorescence microscope (VS120, Olympus) with a 20× objective lens. The number of Fos-, NeuN-, and double-labeled cells was quantified in a blind fashion according to our previous studies (Xue et al., 2017; Fang et al., 2018). In brief, we selected at least three slices for each brain region of each rat and averaged the proportions of neurons expressing Fos on either side of the specific brain region to be the percentage of neuronal activation for each rat and measured the number of Fos-, NeuN-, and double-labeled cells using Image-Pro Plus software.

### Statistical Analysis

All of the data were expressed as mean ± standard error of the mean (SEM) and analyzed using analysis of variances (ANOVAs) with appropriate between- and within-subjects factors (see the “Results” section). Shapiro–Wilk’s test was applied to check normal distribution and Levene’s test was applied to check homogeneity of variance. Tukey’s test was used to conduct *post hoc* analyses of significant effects when prior ANOVAs indicated significant main or interaction effects (*p* < 0.05). *p* < 0.05 was considered to be statistically significant.

## Results

### Experiment 1: Effect of the CS Memory Retrieval–Extinction Procedure on Reinstatement of Methamphetamine Seeking

**Figure 1:**
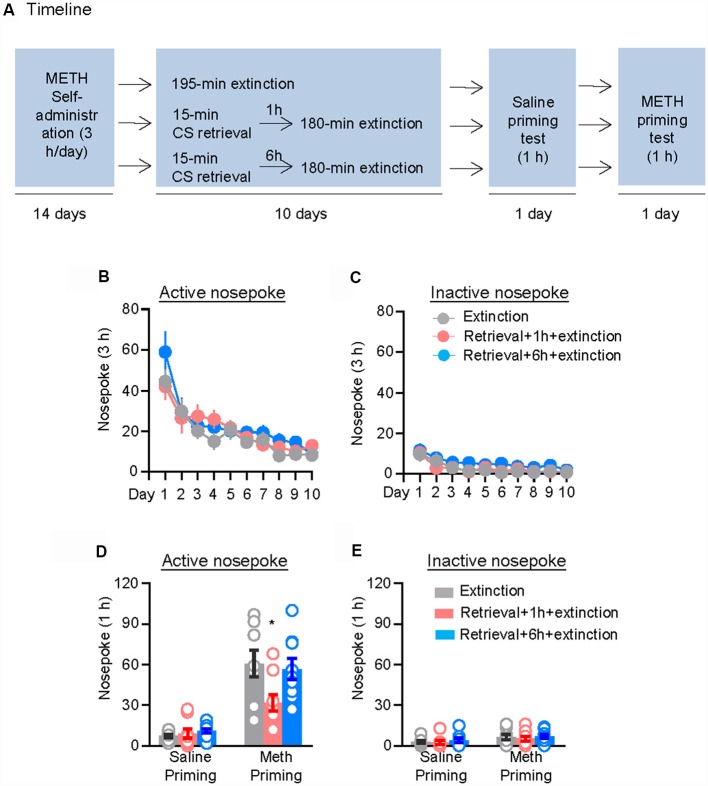
Memory retrieval–extinction procedure prevented drug-priming-induced reinstatement of methamphetamine seeking. **(A)** Experimental timeline. **(B–E)** Nosepokes [mean ± standard error of the mean (SEM)] on the active and inactive nosepoke operandi during the extinction sessions, the saline-priming test, and the methamphetamine (METH)-priming test. *Different from the “Extinction” group; *p* < 0.05; *n* = 8–9 rats per experimental condition.

We first assessed the effect of CS memory retrieval–extinction manipulation on drug-priming-induced reinstatement of methamphetamine seeking. The rats were trained to nose poke for intravenous methamphetamine infusion for 3 h per day for 14 days, after which they were divided into three groups (*n* = 8–9 per group) with equivalent methamphetamine intake for each group and treated as follows: (1) in Group 1, rats received 3.25 h extinction without CS memory retrieval (no retrieval + extinction); (2) in Group 2, rats were given a 15-min CS memory retrieval 1 h before each 3-h extinction session (retrieval + 1 h + extinction); and (3) in Group 3, rats were given CS memory retrieval 6 h before each 3-h extinction session (retrieval + 6 h + extinction). Once the rats met the extinction criterion, they underwent a priming test initiated by a non-contingent methamphetamine injection (1 mg/kg, i.p.) immediately before the test (Figure 1A).

Repeated ANOVA was used to analyze the nosepokes during extinction, with the between-subjects factor of Retrieval–extinction Strategy (no retrieval + extinction, retrieval + 1 h + extinction, and retrieval + 6 h + extinction) and the within-subjects factor of Extinction Sessions (session 1–session 10), and we found significant effect of Extinction Sessions (*F*_(9,207)_ = 23.98, *p* < 0.01, Figure 1B) but no Retrieval–extinction Strategy × Extinction Sessions interactions (*F*_(18,207)_ = 0.87, *p* > 0.05) on active nosepoke operandum. No group difference was observed in nosepokes on the inactive nosepoke operandum (*F*_(18,207)_ = 0.49, *p* > 0.05, Figure 1C). The analysis of the nosepokes during reinstatement test included the between-subjects factor of Retrieval–extinction Strategy (no retrieval + extinction, retrieval + 1 h + extinction, and retrieval + 6 h + extinction) and the within-subjects factor of Test Condition (last extinction session and reinstatement test session). There were significant Retrieval–extinction Strategy × Test Condition interactions on active nosepoke operandum (*F*_(2,23)_ = 3.87, *p* < 0.05, Figure 1D). *Post hoc* analysis showed that in the reinstatement test, the active nosepoke responses significantly decreased compared with the other two groups (*p* < 0.05). No group difference was observed in nosepokes on the inactive nosepoke operandum (*F*_(2,23)_ = 0.08, *p* > 0.05, Figure 1E). These results indicated that exposing rats to the CS retrieval manipulations 1 h but not 6 h before the extinction sessions attenuated methamphetamine-priming-induced reinstatement of methamphetamine seeking.

### Experiment 2: Effect of the CS Memory Retrieval–Extinction Procedure on Spontaneous Recovery of Methamphetamine Seeking

**Figure 2:**
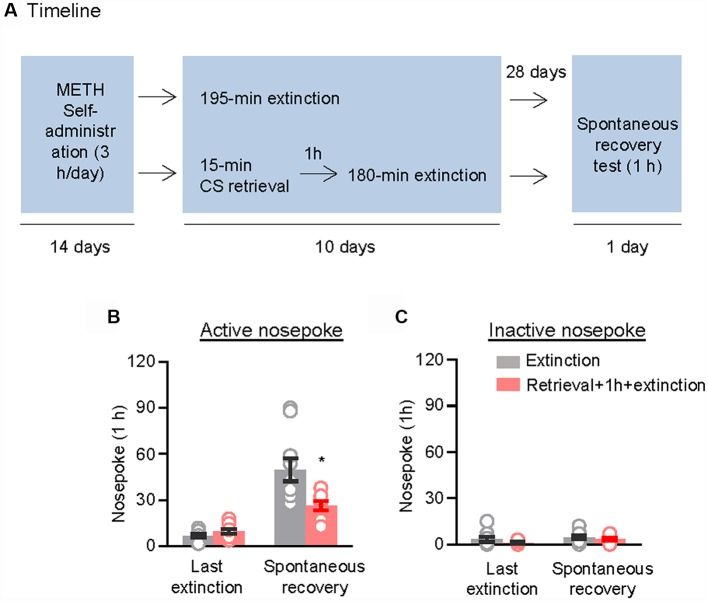
Memory retrieval–extinction procedure prevented spontaneous recovery of methamphetamine seeking. **(A)** Experimental timeline. **(B,C)** Number of nosepokes (mean ± SEM) on the active and inactive nosepoke operandi during the first hour of the last 3-h extinction session and the spontaneous recovery test. *Different from the “Extinction” group; *p* < 0.05; *n* = 9–10 rats per experimental condition.

We used the other two groups of rats to examine the effect of the CS memory retrieval–extinction procedure on spontaneous recovery of methamphetamine seeking. After 14 consecutive days of methamphetamine self-administration training, the rats were divided into two groups (*n* = 9–10 per group) that were matched for their methamphetamine intake: (1) in Group 1, rats underwent 3.25-h extinction training without CS memory retrieval (no retrieval + extinction); and (2) in Group 2, rats were given CS memory retrieval 1 h before each 3-h extinction session (retrieval + 1 h + extinction). When the rats met the extinction criterion, they were housed in their homecages for 4 weeks. Then, they were tested for spontaneous recovery of the extinguished drug-seeking behavior in an extinction session in which nosepokes led to contingent delivery of the tone-light cue previously paired with methamphetamine infusions (Figure 2A).

Repeated ANOVA was used to analyze nosepokes during the spontaneous recovery test, with the between-subjects factor of Retrieval–extinction Strategy (no retrieval + extinction and retrieval + 1 h + extinction) and the within-subjects factor of Test Condition (last extinction session and spontaneous recovery test session). The analysis showed significant interactions between Retrieval–extinction Strategy and Test Condition (*F*_(1,17)_ = 10.58, *p* < 0.01, Figure 2B). No group difference was observed in nosepokes on the inactive nosepoke operandum (*F*_(1,17)_ = 0.30, *p* > 0.05, Figure 2C). The results indicated that exposing rats to the CS retrieval manipulations 1 h before the extinction sessions prevented spontaneous recovery of methamphetamine seeking.

### Experiment 3: Effect of the CS Memory Retrieval–Extinction Procedure on Renewal of Methamphetamine Seeking

**Figure 3:**
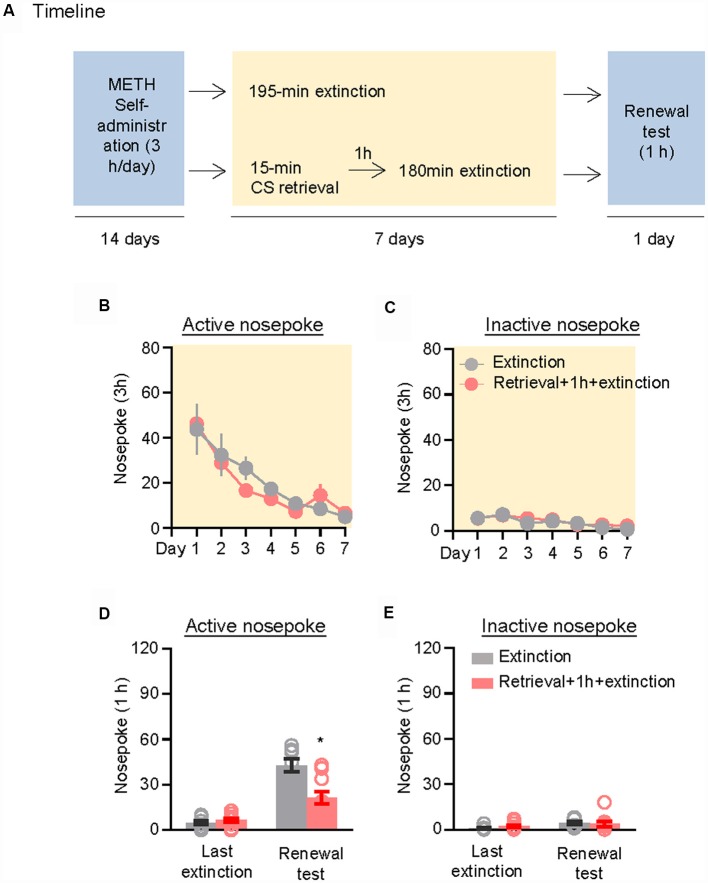
Memory retrieval–extinction procedure prevented renewal of methamphetamine seeking. **(A)** Experimental timeline. **(B–E)** Number of nosepokes (mean ± SEM) on the active and inactive nosepoke operandi during the first hour of the last 3-h extinction session and the renewal test. *Different from the “Extinction” group; *p* < 0.05; *n* = 8–10 rats per experimental condition.

We further demonstrated the effect of the CS memory retrieval–extinction procedure on renewal of methamphetamine seeking under a modified ABA renewal (training in context A, extinction in context B, testing in context A) procedure based on previous studies (Xue et al., 2012; Luo et al., 2015), with two counterbalanced contexts: context A had stainless steel rod floor and gray walls, while context B had granular flat floor and walls covered in wallpaper with black and white patterns. Rats were first trained to self-administer methamphetamine in context A for 14 days. Then, they were divided into two groups (*n* = 8–10 per group) with matched methamphetamine intake and underwent extinction training in context B: (1) in Group 1, rats underwent 3.25-h extinction training without CS memory retrieval (no retrieval + extinction); and (2) in Group 2, rats were given CS memory retrieval 1 h before every 3-h extinction session (retrieval + 1 h + extinction). The CS memory retrieval manipulation was a 15-min exposure to context B, during which nosepokes led to presentation of discrete cues but not methamphetamine. After the rats met the extinction criterion, they underwent a renewal test in context A, during which nosepokes led to contingent delivery of tone-light cues previously paired with methamphetamine infusions but not methamphetamine (Figure 3A).

Repeated ANOVA was used to analyze the nosepokes during extinction, with the between-subjects factor of Retrieval–extinction Strategy (no retrieval + extinction, retrieval + 1 h + extinction) and the within-subjects factor of Extinction Sessions (session 1–session 7). There was only significant effect of Extinction Sessions (*F*_(6,96)_ = 21.26, *p* < 0.01, Figure 3B) but no Retrieval–extinction Strategy × Extinction Sessions interactions (*F*_(6,96)_ = 0.78, *p* > 0.05) on active nosepoke operandum. No group difference was observed in nosepokes on the inactive nosepoke operandum (*F*_(6,96)_ = 0.29, *p* > 0.05, Figure 3C). Repeated ANOVA was used to analyze the nosepokes during renewal test, including the between-subjects factor of Retrieval–extinction Strategy (no retrieval + extinction, retrieval + 1 h + extinction) and the within-subjects factor of Test Condition (last extinction session, renewal test session). There were significant Retrieval–extinction Strategy × Test Condition interactions on active nosepoke operandum (*F*_(1,16)_ = 14.74, *p* < 0.01, Figure 3D). No group difference was observed in responding to the inactive nosepoke operandum (*F*_(1,16)_ = 1.52, *p* > 0.05, Figure 3E). The results indicated that exposing rats to the CS retrieval manipulations 1 h before the extinction sessions prevented subsequent renewal of methamphetamine seeking.

### Experiment 4: Effect of the CS Memory Retrieval–Extinction Procedure on Neuronal Activation in Amygdala

**Figure 4:**
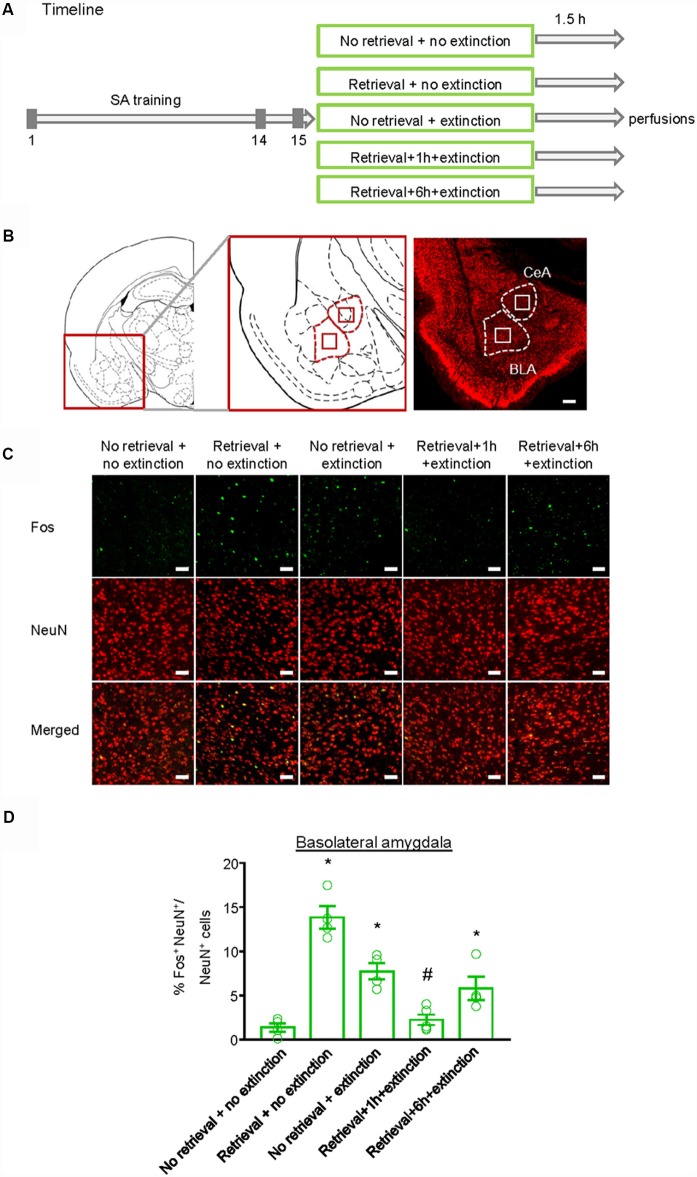
Effect of the conditioned stimulus (CS) retrieval–extinction procedure on neuronal activation in basolateral amygdala (BLA). **(A)** Experimental timeline. Rats were trained to self-administer methamphetamine during three 1-h daily sessions over 14 days. One day later, rats were divided into five experimental groups and were perfused 1.5 h after the treatment. **(B)** Coronal section schematic indicating the region of BLA and central amygdala (CeA) with NeuN immunofluorescence staining. Scale bar is 200 μm. **(C)** Representative images showing green (Fos protein), red (NeuN protein), and double-labeled neurons in the BLA in different experimental manipulations. Scale bars represent 50 μm. **(D)** Percentage of activated cells in the BLA in different experimental conditions. The CS memory retrieval–extinction manipulation with a 1-h but not 6-h interval attenuated neuronal activations in the BLA. *n* = 4–5 per experimental condition. Data are mean ± SEM of number of overlap (Fos + NeuN protein-IR/Fos-IR). *Different from the “No retrieval + no extinction” group. ^#^Different from the “No retrieval + extinction” group, one-way analysis of variance (ANOVA), *p* < 0.05.

**Figure 5:**
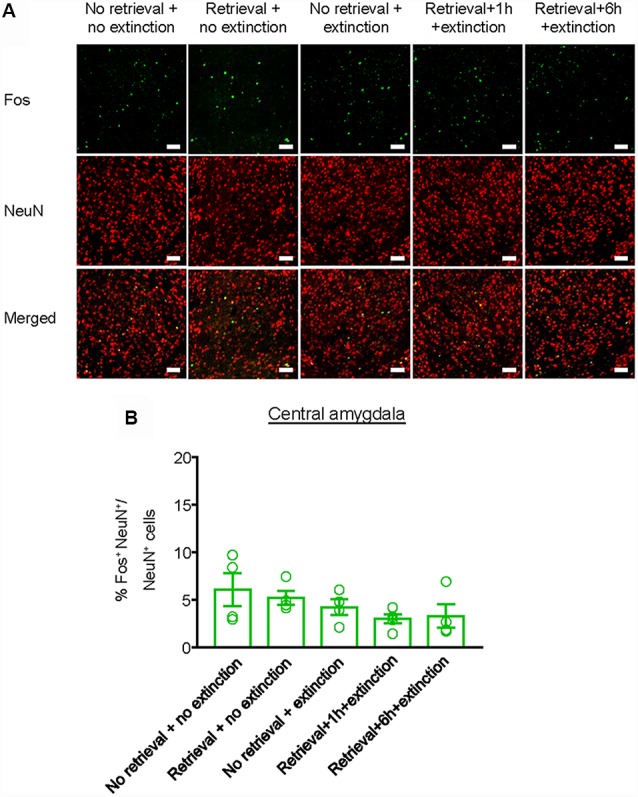
Effect of the memory retrieval–extinction procedure on neuronal activation in the CeA. **(A)** Representative images showing green (Fos protein), red (NeuN protein), and double-labeled neurons in the CeA in different experimental manipulations. Scale bars represent 50 μm. **(B)** Percentage of activated cells in the CeA in different experimental conditions. The memory retrieval–extinction manipulation with a 1-h interval did not affect neuronal activations in the CeA compared with the extinction group. *n* = 4–5 per experimental condition. Data are mean ± SEM of number of overlap (Fos + NeuN protein-IR/Fos-IR).

Finally, we investigated whether the inhibitory effect of the CS memory retrieval–extinction procedure on reinstatement of methamphetamine seeking was associated with neuronal activation in BLA and central amygdala (CeA), two subregions of amygdala (LeDoux, 2007). Fos was widely used as the marker of neural activations (Morgan and Curran, 1991; Xue et al., 2017; Luo et al., 2018; Venniro et al., 2018), and it has been found to mediate memory reconsolidation (Miller and Marshall, 2005). Thus, we assessed whether Fos expression was different for standard extinction, extinction within the time window of reconsolidation, and extinction out of the time window of reconsolidation. Rats were first trained to self-administer methamphetamine for 14 days. One day later, they were divided into five groups (*n* = 4–5 per group) with matched methamphetamine intake and perfused following different manipulations (Figure 4A): (1) in Group 1, rats did not undergo either CS memory retrieval or extinction training (no retrieval + no extinction); (2) in Group 2, rats only underwent CS memory retrieval (retrieval + no extinction); (3) in Group 3, rats underwent 3.25-h extinction training without CS memory retrieval (no memory retrieval + extinction); (4) in Group 4, rats were given CS memory retrieval 1 h before the 3-h extinction session (retrieval + 1 h + extinction); and (5) in Group 5, rats were given CS memory retrieval 6 h before the 3-h extinction session (retrieval + 6 h + extinction). The rats were perfused 1.5 h after different manipulations and their brains were removed for immunofluorescence assays to assess the co-expression of Fos and NeuN (a marker of neurons) in the BLA (Figures 4B,C) and CeA (Figure 5A). One-way ANOVA was used to analyze immunofluorescence data, revealing the significant effect of Retrieval–extinction Strategy (*F*_(4,16)_ = 26.96, *p* < 0.01, Figure 4D). *Post hoc* analysis showed that, compared with the no retrieval group, both retrieval + no extinction and no retrieval + extinction groups increased the Fos expression (*p* < 0.01). More importantly, the CS memory retrieval–extinction manipulation with a 1-h but not 6-h interval attenuated neuronal activation in the BLA compared with the extinction group (*p* < 0.01). In contrast, no significant difference in Fos expression was found in the CeA of all groups (*F*_(4,16)_ = 1.56, *p*-values > 0.05, Figure 5B), indicating that the changes in Fos expression induced by CS memory retrieval–extinction was specific to BLA.

## Discussion

In the current study, we demonstrated that the memory retrieval–extinction procedure could be an effective method to prevent methamphetamine seeking and relapse. Daily extinction training 1 h after CS retrieval significantly attenuated drug-priming-induced reinstatement of methamphetamine seeking. In addition, the memory retrieval–extinction procedure reduced relapse following 28 days of abstinence, indicating its long-lasting effect on drug addiction. In an ABA renewal model, the memory retrieval–extinction procedure also impaired the context-induced reinstatement of methamphetamine seeking. Despite the fact that the memory retrieval–extinction procedure was proved effective in preventing drug seeking and relapse in both animals and human addicts (Ma et al., 2012; Xue et al., 2012; Millan et al., 2013; Sartor and Aston-Jones, 2014; Germeroth et al., 2017), the neural mechanisms involved in this process remains largely elusive. Here, we found that presenting extinction within the time window of reconsolidation, but not presenting extinction out of the time window of reconsolidation or standard extinction training inhibited retrieval-induced neuronal activation in the BLA. Taken together, these results revealed that memory retrieval–extinction manipulations could potentially be utilized for the treatment of methamphetamine addiction, and its efficacy may be related to engagement of BLA.

The development of a memory retrieval–extinction procedure was based on the theory of reconsolidation as well as pharmacological studies targeting memory retrieval and reconsolidation process (Lee et al., 2006; Milekic et al., 2006; Milton et al., 2008b; Li et al., 2010; Sanchez et al., 2010; Wouda et al., 2010). Reconsolidation is defined as a process during which a stabilized memory turns into a labile phase induced by memory retrieval and thus can be modified (Sorg, 2012; Forcato et al., 2014; Nader, 2015; Lee et al., 2017). Increasing evidence suggested that only pharmacological or behavioral manipulations within a limited time interval after memory retrieval could disrupt memory reconsolidation, indicating the existence of a reconsolidation time window (Walker et al., 2003; Li et al., 2010; Schiller et al., 2010; Xue et al., 2012; Luo et al., 2015). In line with previous studies, we found that extinction training performed 6 h after memory retrieval failed to prevent the drug-priming-induced reinstatement of methamphetamine seeking.

Considering that the memory retrieval manipulation was performed within the consolidation window of extinction memory, an alternative explanation for the effect of retrieval–extinction procedure could be that the extinction memory was facilitated, leaving the original rewarding memory less susceptible to reinstatement, spontaneous recovery, and renewal. In fact, pharmacological interventions that facilitated consolidation of extinction successfully attenuated the reinstatement, spontaneous recovery, and renewal of fear response and drug seeking (Quirk and Mueller, 2008; Malvaez et al., 2010). Nevertheless, the reduction in methamphetamine seeking and relapse does not seem to be related with enhanced extinction, considering the comparable rate of extinction among different groups.

In recent years, there has been some progress in uncovering the neural substrates of the effect of memory retrieval–extinction procedure on memories (Cahill and Milton, 2019). For example, Monfils et al. (2009) showed that a second CS presented 1 h after the initial retrieval led to dephosphorylation of GluA1 in the lateral amygdala, which may underlie the disappearance of fear reemergence in the retrieval–extinction paradigm. Consistently, Clem and Huganir (2010) found that Ser845 mutant mice did not show reduction in fear reemergence after the retrieval–extinction procedure. Moreover, they found synaptic removal of CP-AMPARs in the lateral amygdala in the retrieval–extinction procedure but not standard extinction (Clem and Huganir, 2010). All of these results suggested that the retrieval–extinction procedure triggered dephosphorylation of the GluA1Ser845 and produced depotentiation of the original memory, rather than enhancement of extinction (Monfils et al., 2009; Clem and Huganir, 2010). Tedesco et al. (2014) found an increase in the expression of zinc-finger 268 protein and phosphorylated ribosomal protein S6 in the prefrontal cortex and lateral amygdala during fear retrieval–extinction compared with extinction alone. They proposed that the effect of the retrieval–extinction process was more similar to reconsolidation updating than extinction facilitation or reconsolidation disruption (Tedesco et al., 2014). Lee et al. (2016) found fewer Arc-staining cells in the lateral amygdala during the late phase retrieval–extinction group. However, in contrast to the relatively more research focusing on the role of retrieval–extinction in fear, knowledge about the neural mechanism underlying the effect of retrieval–extinction on drug addiction remains scarce. In the current study, we examined the changes of Fos, one of the immediate early genes that is involved in processes of reconsolidation and extinction (Miller and Marshall, 2005; Siahposht-Khachaki et al., 2017, 2018), after retrieval, extinction, and retrieval–extinction procedures. We found that Fos expression increased after retrieval and extinction, consistent with previous studies (Nic Dhonnchadha et al., 2012; Xue et al., 2017). The increased activity of BLA neurons detected after extinction might indicate the extinction memory, i.e., a new inhibitory memory of CS-no reward. For the groups of “retrieval + 1 h + extinction” and “retrieval + 6 h + extinction,” the activity of BLA neurons might indicate the effects of updating the original “CS-reward” trace with a new “CS-no reward” trace within or outside the reconsolidation time window, respectively. Presenting extinction 1 h after retrieval decreased Fos expression to the level of no retrieval. It is interesting that the phenomena did not occur in the standard extinction or extinction 6 h after retrieval. These results suggest that decreased Fos expression may not be due to the differences in time between first re-exposure to the chamber and perfusion, but reflect the interaction between retrieval and extinction, i.e., interference of reconsolidation by extinction or interference of extinction by reconsolidation. The boundary conditions and interaction of reconsolidation and extinction abolished the neuronal activation and Fos expression and may weaken the memory engram of addiction memory in BLA. In addition, there was no difference among these groups in the CeA. Consistently, our previous study has shown that the memory retrieval–extinction procedure with a 10-min but not 6-h interval or standard extinction decreased PKMζ expression in the BLA (Xue et al., 2012). Together with previous studies in fear memory, these findings suggested that the retrieval–extinction procedure involves mechanisms that differ from standard extinction. Further studies combining optogenetics and electrophysiology are warranted to elucidate the causal role of BLA in the memory retrieval–extinction procedure. In addition, BLA has been considered to have intricate connections with the prefrontal cortex, hippocampus, and nucleus accumbens, as well as sensory association areas (Brog et al., 1993; McDonald, 1998; Ghashghaei and Barbas, 2002; Ghashghaei et al., 2007). Future studies should investigate how BLA interacts with other brain areas to mediate the effect of memory retrieval–extinction on drug seeking.

In summary, we showed significant inhibitory effects of the memory retrieval–extinction procedure on drug-priming-induced reinstatement, spontaneous recovery, and renewal of methamphetamine seeking in rats, suggesting that it could be a promising method for decreasing relapse in methamphetamine addicts. Furthermore, the attenuated neuronal activation in the BLA and disrupted memory reconsolidation may be associated with the favorable behavioral outcomes of the memory retrieval–extinction procedure.

## Ethics Statement

All of the experimental procedures were performed in accordance with the National Institutes of Health Guide for the Care and Use of Laboratory Animals and were approved by the Biomedical Ethics Committee on animal use and protection of Peking University.

## Author Contributions

Y-YC, L-BZ, Y-XX, and LL designed the experiments. Y-YC, L-BZ, and YL performed the experiments. Y-YC, L-BZ, and Y-XX analyzed and interpreted the data. S-QM, Y-MG, and JS commented on the manuscript. Y-YC and Y-XX wrote the manuscript.

## Conflict of Interest Statement

The authors declare that the research was conducted in the absence of any commercial or financial relationships that could be construed as a potential conflict of interest.
